# Polygenic risk scores in cancer

**DOI:** 10.1515/medgen-2026-3011

**Published:** 2026-07-08

**Authors:** Anja Tüchler, Laura Grohs, Alexander Volk, Stefan Aretz

**Affiliations:** Institute for Hereditary Cancers Center for Integrated Oncology (CIO), Faculty of Medicine Cologne Germany; University of Cologne University Hospital Cologne Kerpener Str. 62 50937 Cologne Germany; University of Bonn Institute of Human Genetics Medical Faculty Bonn Germany; University Hospital Bonn National Center for Hereditary Tumour Syndromes Venusberg-Campus 1 53127 Bonn Germany; Institute for Hereditary Cancers Center for Integrated Oncology (CIO), Faculty of Medicine Cologne Germany; University of Cologne University Hospital Cologne Kerpener Str. 62 50937 Cologne Germany; University of Bonn Institute of Human Genetics Medical Faculty Bonn Germany; University Hospital Bonn National Center for Hereditary Tumour Syndromes Venusberg-Campus 1 53127 Bonn Germany

**Keywords:** Tumour predisposition syndromes, risk stratification, prevention, surveillance, genetic diagnostics

## Abstract

Polygenic risk scores (PRS) have emerged as a powerful tool to refine cancer risk prediction beyond age, family history, and modifiable environmental risk factors, enabling individualized prevention and early detection strategies. Evidence from breast, colorectal, and prostate cancer demonstrates that PRS significantly improve risk stratification in both sporadic and hereditary settings and can modify penetrance of pathogenic germline variants. Integrating PRS into multifactorial models such as CanRisk enhances predictive accuracy and supports risk-adapted preventive approaches. While implementation studies show feasibility, prospective validation and standardization as well as ancestry-specific calibration remain key challenges for clinical translation.

## Introduction

Cancer represents a major global public health burden. Non-genetic risk factors include age, family history (FH), and lifestyle / environmental exposures. Moderate and high-penetrant pathogenic germline variants (PGV) underlie a plethora of specific monogenic tumour disposition syndromes.

For some cancer types, primary and secondary preventive approaches are recommended for the general population and certain high-risk groups, which prevent cancer or result in early detection, some of which have been proven to efficiently reduce morbidity and mortality. However, in current practice, risk prediction and risk-based surveillance recommendations are still based on a limited number of risk factors, in particular age. Such an approach does not account for the wide variation in individual cancer risks and disregards younger people with a higher risk, while it may overtreat those with low risks.

**Figure 1:**
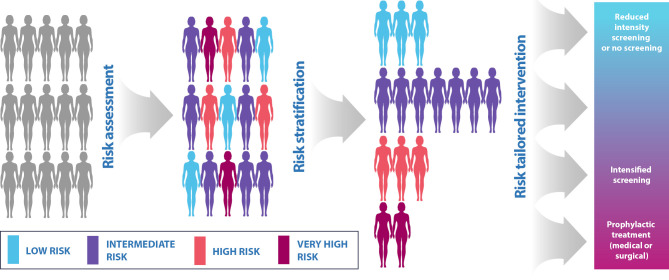
Risk stratification approach for cancer prevention *(Figure by Pashayan et al. [55], used unter Commons Attribution 4.0 International License [*https://creativecommons.org/licenses/by/4.0/*]. No changes made.)*

Accurate cancer risk prediction models are critical for identifying individuals at low and high risk to offer individualized, risk-stratified preventive interventions with more intensive surveillance in high-risk groups and less interventions in low-risk groups (Fig. 1). Cancer-associated polygenic risk scores (PRSs), calculated from the aggregated effects of multiple common single nucleotide polymorphisms (SNPs), represent powerful and independent modifiers of cancer risk for both sporadic and hereditary types of cancer, which can improve risk stratification and inform tailored preventive approaches. It is supposed that a high polygenic risk, defined as the highest PRS quintile, may explain 4–30 % of incident cancer cases, which exceeds the estimates for other risk factors for several cancers [29]. The most important benefit of genetic risk prediction is the identification of high-risk individuals who would otherwise not be identified. In this article, we summarise the present status of PRS-based risk prediction in oncology with a focus on three common and most relevant cancers for its short-term implementation in routine care.

## Breast cancer

Among solid tumors, breast cancer (BC) represents a leading example for the clinical translation of PRSs, both in genetic risk assessment for hereditary breast and ovarian cancer (HBOC) and in ongoing efforts to implement risk-stratified population-based mammography screening. One in eight women will be diagnosed with BC in the course of her life, with around 75,000 diagnoses in Germany each year. Age is a common risk factor for BC – but 15 % of all BC diagnoses affect women under the age of 50 years [58].

Early detection plays an important role in BC prevention, but currently, mammography screening programmes are typically based on age alone. In 2019, the European Collaborative on Personalized Early Detection and Prevention of Breast Cancer (ENVISION) emphasized the need for risk-stratified screening approaches, in which individual BC risk is assessed and women are allocated to tailored prevention and screening strategies (Fig. 1). Such personalized programmes aim to maximize the benefits of early detection while minimizing harms such as overdiagnosis and unnecessary interventions [55]. One approach for risk stratification is the implementation of a BC-based PRS in risk assessement.

Genome-wide association studies (GWAS) conducted within the Breast Cancer Association Consortium (BCAC) have enabled the development of several BC-based PRS models. The most widely used is the BCAC PRS comprising 313 SNPs (or subsets thereof), derived from a dataset of 94,075 cases and 75,017 controls of European ancestry [46].

For overall BC, the BCAC313 PRS is associated with an OR of 1.61 (95 % CI, 1.57 to 1.65) per SD increase and provides moderate discrimination (AUC 0.63) [32, 46]. For the general population, the authors estimate that 35 % of all BC cases would be attributed to women in the highest PRS quintile – whereas 9 % of cases would occur in women in the lowest quintile [46].

### Familial and hereditary breast cancer: primary and secondary prevention

**Figure 2:**
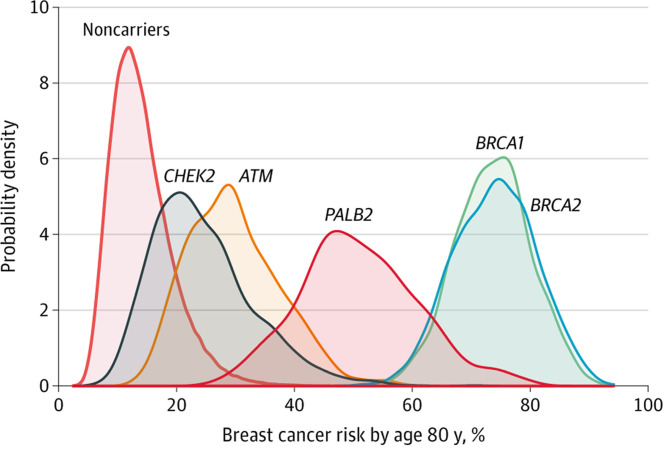
Probability density function displaying the modification of absolute breast cancer risk estimates by 80 years for women carriers of pathogenic germline variants (PGV) in high- and moderate-risk breast cancer genes and non-carriers. (Figure by Gallagher et al. [17]. Courtesy of Mark Robson, MD, Memorial Sloan Kettering Cancer Center, 1275 York Ave, New York, USA)

A modifying effect of the PRS also has been demonstrated among carriers of PGVs in *BRCA1* and *BRCA2*, where PRSs can lead to considerable risk stratification in predicted lifetime and contralateral BC risk [17, 31, 33]. Among carriers and non-carriers of PGVs in non-*BRCA1*/*2* genes (e.g., *PALB2*, *ATM*, *CHEK2*), which are associated with varying levels of penetrance, PRS enable risk stratification that is assumed to be more pronounced than the PRS’ effect on risk stratification in PGVs in *BRCA1*/*2,* leading to considerable changes in predicted BC risk (Fig. 2) [4, 17, 34]. It is current best practice to integrate an individual’s PRS into multifactorial risk models, such as the BOADICEA-algorithm implemented in the CE-certified CanRisk tool [37]. Model performance improves, when the PRS is considered alongside additional risk factors such as mammographic breast density, hormonal factors, and FH of cancer (AUC 0.66, 95 % CI 0.66-0.67 [57]). The consideration of PRSs in risk calculation has been shown to lead to changes in clinical recommendations [34, 66], but studies on its clinical utility in terms of improved effectiveness of HBOC screening, efficiency, and health-economic benefit are lacking.

For PGV carriers in *BRCA1*/*2*, PRS-refined BC risks can support risk communication in non-directive genetic counselling and may guide discussion on women’s risk-management strategies, including age of onset of intensified breast surveillance and the option to undergo prophylactic surgery (i.e., bilateral or contralateral risk-reducing mastectomy) [44, 66].

In carriers of PGVs in moderate BC susceptibility genes, current guidelines generally recommend intensified breast surveillance with varying screening modalities across Europe [44], while in general, risk-reducing mastectomy is not indicated. In singular cases, the PRS-informed BC risk calculation may identify individuals at the upper PRS tail end, where risk-reducing mastectomy (both contralateral andbilateral) may be discussed as an alternative to intensified breast surveillance. However, this should only be considered on a case-by-case basis and require careful consideration of BC risks (including BC FH and non-genetic risk factors), competing risks and age.

### Secondary prevention in the general-population: population-based screening programmes

Several international studies are currently investigating the utility of individualized risk assessment including BC PRSs in population-based mammography screening programmes. With the aim of offering risk-adapted BC screening, these approaches seek to offer women at higher risk more frequent screening intervals (e.g., annual mammography) or enable the de-escalation of screening intensity for those at lower risk (e.g., mammography intervals every four years) [12, 56]. Early results suggest that risk-adapted BC screening in the general population did not lead to a reduction in biopsy rates, but is safe and has little to no negative psychological impact on patient-reported outcomes [12, 56]. However, model studies considering FH and PRS for BC risk assessment suggest that risk-adapted screening strategies may lead to increased rates of overdiagnosis and false positive screening results, potentially leading to additional diagnostic procedures such as imaging follow-up or biopsy [67].

While the studies primarily aim to evaluate the effectiveness of such risk-adjusted programmes and the clinical utility of PRS-informed BC risk stratification, they also address key implementation challenges of risk-adapted screening, such as feasibility and acceptance [59]. The German myRisk study, funded by the German Cancer Aid, focuses on aspects such as compliance with the Gene Diagnostics Act (Gendiagnostikgesetz, GenDG), the cost-effective computation of PRS, and the development of comprehensible patient information materials.

## Colorectal cancer

Colorectal cancer (CRC) is the second (women) and third (men) most common cancer in Germany with an annual incidence of around 55,000 [58]. Established risk factors include age, Western life style, colorectal polyps, a positive FH of polyps and CRC including early-onset disease, monogenic (hereditary) types of CRC, in particular Lynch syndrome (LS), and few other specific conditions, while moderate penetrant risk factors beyond *PMS2* and specific *APC* germline variants seem hardly to exist. The majority of familial and early-onset CRC (EO-CRC) cannot be explained by monogenic subtypes and is supposed to result from a multifactorial or polygenic etiology [9].

Fortunately, CRC is among the most preventable cancers, in part because CRC screening is effective for both early detection of treatable cancers and for reducing cancer risk by removing pre-cancerous lesions (polyps) [5]. In particular, colonoscopies can drastically reduce CRC risk in both the general population and high risk groups [6]). However, a comprehensive risk stratification with tailored, individualized surveillance is still evolving. According to the updated German S3 guideline CRC, several risk groups have been defined with specific surveillance recommendations (Tab. 1) [39].

A genetic disposition beyond the 3–5 % monogenic types contributes significantly to the CRC burden, since 16 %–35 % of interindividual variability in CRC risk has been attributed to inherited factors based on twin and family studies [42]. Common disease-associated SNPs are supposed to represent a relevant fraction of the estimated overall heritability. In the last two decades,, more than 90 independent loci with hundreds of common, low-penetrant CRC-associated SNPs have been identified by GWAS [15, 23, 35, 60]. Subsets of those variants were used to derive CRC-associated PRSs and estimate their impact on CRC risk modulation. Although the studies differ in terms of selected SNPs (31–204), PRS models [1, 48, 64], included risk factors, patient cohorts, recruitment strategy, and overall study design, it could be demonstrated consistently, that the polygenic background exhibits significant risks of developing CRC both in the general population and specific risk groups including LS.

### CRC-associated PRS in the general population

**Figure 3:**
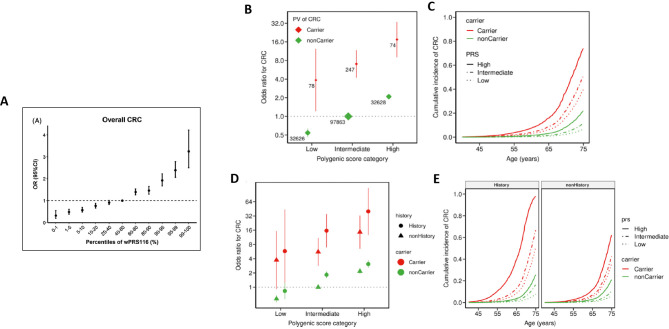
Colorectal cancer (CRC) odds ratio and cumulative incidence stratified by carrier and family history status. **(A)** Odds ratios and 95 % confidence intervals for associations between the percentiles of wPRS116 and site-specific CRC risk in the UK Biobank. wPRS116: weighted polygenic risk score of 116 CRC SNPs *(Figure by Li et al. [42]; used unter Commons Attribution 4.0 International License [*https://creativecommons.org/licenses/by/4.0/*]. No changes made).*** (B + C)** Individuals from UK Biobank were stratified for PGV carrier status in the Lynch syndrome genes (*MLH1, MSH2, MSH6*, or *PMS2*) into three strata based on their PRS: Low (< 20 % percentile), intermediate (20–80 % percentile), or high (> 80 % percentile) PRS. The odds ratio (OR) was calculated from a logistic regression model with age, sex, CRC screening status, and the first four principal components of ancestry as covariates. The reference group was non-carriers with intermediate PRS (B). The adjusted OR is indicated by the colored boxes. The numbers next to the ORs indicate the sample size of the corresponding group. The 95 % CIs are indicated by the vertical lines around the boxes. Cumulative incidence was estimated from a cox-proportional hazard model using age, sex, family history, CRC screening status, and the first four ancestry principal components as covariates. **(D + E)** Interplay of PGV carrier status, family history, and PRS; non-carriers with intermediate PRS and no family history served as the reference group. *(Figures by Hassanin et al. [19]; used unter Commons Attribution 4.0 International License [*https://creativecommons.org/licenses/by/4.0/*]. No changes made apart from numbering of the figures)*.

PRS studies since 2015 demonstrated that the risk of CRC is modified considerably by a polygenic component, both in terms of age at onset and cumulative lifetime risks [22, 30, 65], although common variants may explain not more than 10 % of the heritable fraction of CRC risk [28]. PRS performance was higher, when LS carriers were excluded from datasets [1]. In summary, those studies found that individuals in the lowest polygenic risk strata (<20 %) exhibit, on average, an approximately two to four fold reduction in CRC prevalence, with those in the lowest 1 % having up to a 70 % lower risk than the mid-quintiles [19, 27]. Conversely, individuals in the PRS top 20 %, top 10 %, and top 1 % have roughly two, three, and four fold higher risks, respectively (Fig. 3A). This corresponds to lifetime risks of around 20 % [19, 42]. Hence, individuals with a very high PRS may have CRC risk levels even comparable to carriers of monogenic conditions [13, 63]. The vast majority of these individuals have no CRC FH and would have been considered average risk under current screening guidelines. Similar PRS-dependent patterns have recently been reported for relevant CRC precursors (advanced adenomas) and post-colonoscopy CRCs [63, 71].

### CRC-associated PRS in high risk groups

Although the CRC lifetime risk can be very high in different risk groups if untreated, a striking intra- and intrafamilial variability was observed. It can be assumed, that the same risk factors that modulate CRC risk in the general population are also relevant in high-risk constellations. Consistent with these assumptions, CRC-associated risk alleles tend to accumulate in unexplained familial and EO (in particular sporadic) CRC cases [2, 51]. Moreover, PRS has been shown to considerably modulate CRC risks in individuals with LS, caused by a PGV in one of the four mismatch repair (MMR) genes or the *EPCAM* gene [11, 19].

However, the impact of the PRS strongly depends on study design, recruitment strategy, and analytical methods [11, 19, 25, 64]. In population-based cohorts (UK Biobank), the estimated absolute cumulative CRC incidence for LS carriers by age 75 ranged from 11–80 % (lowest to highest PRS percentile) and 40–74 % (lowest and highest PRS quintile), respectively [13, 19] (Fig. 3, B-E). As expected, the modifiying effect of the PRS is negatively correlated with the penetrance of the MMR gene, so that the largest impact was seen for *PMS2* (the gene with the lowest penetrance of all MMR genes) followed by *MSH6*, similar to what is known for moderate penetrant BC genes [19]. This suggests that PRS inclusion in risk stratification may be particular relevant to carriers of PGV in moderate penetrant genes.

In clinically ascertained LS cohorts, however, strong polygenic effects were not observed [11, 25]. It is likely that this discrepancy is mainly caused by the very different relative contribution of the four MMR genes: while cohorts with LS carriers recruited due to fullfilled clinical criteria, are enriched for PGV in high-penetrant genes (*MLH1, MSH2*), the vast majority of population-based LS patients carry PGV in low-to moderate penetrant genes (in particular *PMS2*). In addition, a familial clustering of CRC might reflect the existence of several genetic and non-genetic risk factors, which are not captured by the PRS and which may superimpose the polygenic impact. Consequently, the application of PRS in clinical practice should consider the familial background and ascertainment of patients.

### Integration of PRS with other risk factors

To most accurrately estimate the actual overall CRC risk for a precise, individualized stratification followed by tailored surveillance strategies, ideally all known risk factors are captured and integrated in comprehensive models. Several studies analysed the effects of various combinations of relevant risk and protective factors (Fig. 3). Although no study included all of them (age, FH, non-genetic risk factors, LS carrier status, PRS, colonoscopy), risk prediction models showed consistently that i) each main risk factor including the PRS, contributes significantly and independently to risk increase or decrease, most of which to a similar extent, and ii) integrated models improve discriminatory accuracy: while the AUC derived from PRS (0.58–0.69) was in general higher compared to those using FH (0.52–0.65), lifestyle (0.59–0.60), or carrier status (0.65), integrated models showed a better prediction (0.63–0.72) [1, 6, 26]. In individuals without FH, but high PRS, the CRC risk is doubled, whereas a low PRS even in the context of a FH results in a decreased risk. Although LS carriers showed a priori a significantly increased CRC risk, both the PRS and FH modify these risks considerably with a variability of the cumulative CRC incidence between 35 and 98 % [19]. As a consequence, modifiable factors (lifestyle, surveillance) can compensate genetic dispositions to a certain degree. In the future, the inclusion of further, CRC-associated rare variants in prediction models may further improve risk prediction.

### Implications – Implementation in clinical care

Several studies have consistently demonstrated that a CRC-associated PRS is an important additional component for stratification into high, intermediate, and low risk groups, which is independent and equivalent to other established factors or even outperform those in terms of discriminatory power. This is not only true for late-onset sporadic cases, but also for EO, familial, and hereditary CRC. Hence, the implementation of a PRS-based risk prediction in routine care will complement other risk factors and should have practical implications for surveillance recommendations in terms of starting age and surveillance intervals. The present data provide a straightforward basis for designing a PRS-based population CRC screening programme, although some substantial barriers have to be solved (see Schumacher et al. “Polygenic risk scores in clinical applications – opportunities and challenges”). Translating relevant risk factor information into actionable clinical information is the next step in developing personalized prevention [26]. To apply a CRC-associated PRS in clinical practice, testing must be complemented by clinical recommendations for preventive activities at different levels of risk.

**Figure 4:**
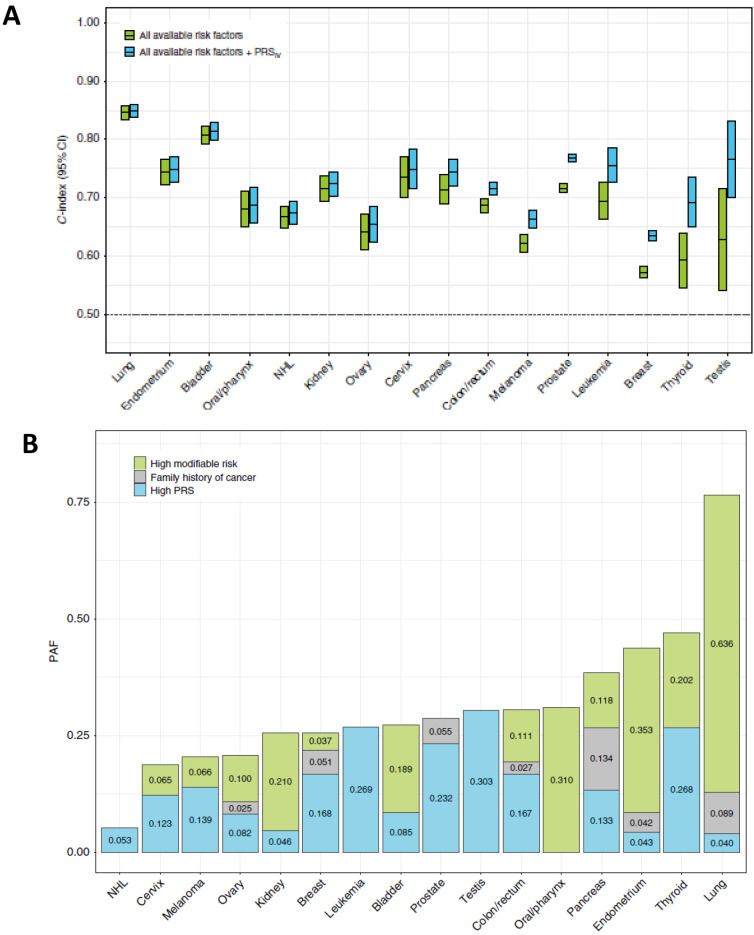
PRS-contribution across various malignancies. **(A)** Assessment of model discrimination based on Harrell’s C-index between 1 and 5 years of follow-up time. Comparisons are conducted between the most comprehensive risk factor model for each cancer, including all available lifestyle-related risk factors and family history (if applicable), and a nested model that also includes the standardized polygenic risk score (PRS_IV_) for that cancer and the top 15 genetic ancestry principal components. **(B)** Population attributable fractions (PAF) estimated at 5 years of follow-up time. PAF estimates for the top 20 % (≥80th percentile) of the modifiable risk factor and PRS distributions, respectively, and family history of cancer, were derived from Cox proportional hazard regression models adjusted for age at enrollment, sex, genotyping array, and the top 15 genetic ancestry principal components *(Figures by Kachuri et al. [29]; used unter Commons Attribution 3.0 IGO License [*https://creativecommons.org/licenses/by/3.0/igo/*]. No changes made apart from numbering of the figures)*.

**Table 1:** Criteria for colorectal cancer risk stratification according to the German S3 guideline Colorectal Cancer [39].

**CRC risk factor / Risk group**	Surveillance / preventive strategy *
**Western Lifestyle** smoking, alcohol, diet, physical activity, body fatness	Improvement of lifestyle No risk-adapted surveillance strategy
**Age** >50 years	Colonoscopy every 10 years or Sigmoidoscopy every 5 years or Immunochemical Fecal Occult Blood Test (iFOBT) every 1–2 years
**Colorectal polyps / polyposis**	Endoscopic surveillance of gastrointestinal tract depending on number, size, histology, dysplasia of polyps / polyposis subtype
**Positive Family history** regarding polyps or CRC without suspected polyposis or hereditary CRC	Colonoscopies at least every 10 years, starting 10 years before earliest manifestation, latest at 40 years of age
**Suspected hereditary CRC in patient / family** EO-CRC, CRC clustering and / or dMMR / MSI in tumour; without causative germline variant	Colonoscopies every 3–5 years, starting 10 years before earliest CRC manifestation, latest at 40 years of age
**Hereditary CRC** (Lynch syndrome, polyposis)	Syndrome-specific interventions (intensive endoscopy, preventive surgery, chemoprevention)
**Other specific conditions** inflammatory bowel diseases (IBD), Cystic fibrosis	Syndrome-specific recommendations
**PRS**	Not yet included in guideline, but integration as additional factor for risk stratification in future versions under discussion

*German S3 guideline “Colorectal Carcinoma” [reference 39]

In Germany, a preventive colonoscopy is recommended for the general population by age 50, when the cumulative CRC incidence is estimated to be around 0.5 % and the prospective absolute 10-year risk around 1 % [26]. A roadmap for a PRS-based CRC risk stratification may include i) identification and validation of the best performing model for a PRS test using data sets from genetic biobanks; ii) application of the PRS model to develop absolute risk-adapted surveillance recommendations based on the average CRC risk level at 50 years currently accepted for CRC public surveillance, iii) routine clinical implementation of the test in prospective pilot studies, and iv) retrospective analysis of the results [54]. Prospective clinical pilot trials are needed to further address the acceptance, feasibility, clinical utility, and cost-effectiveness of PRS-based risk programmes.

The vast majority of the 20 % of individuals in the general population with a doubled CRC risk based on a PRS in the high quintile (in Germany roughly 14 out of 70 million adult people), which is similar to the risk of individuals with a first degree relative affected by CRC, has no positive FH and thus is otherwise non-recognisable as target group for advanced CRC surveillance beginning at around 40 years. In contrast, a substantial fraction of individuals even with a positive FH can delay the start of screening to age 50 or beyond based on a PRS in the low quintile. Considering the extreme ends of the PRS in those with no FH, the range of recommended age to start CRC surveillance can be as wide as 20 years for individuals at the top 1 % and bottom 1 % [26].

Since risk stratification based on age, FH, and monogenic conditions is already recommended and partly established, the inclusion of a PRS as additional risk predictor should be feasible. Unlike lifestyle factors, which vary during life course, the PRS provides a constant value that could be measured once at a relatively young age, such as 40 years (or even younger and coupled with screening for high penetrant hereditary CRC PGV), and then be used for personalized advice on when to start screening [71]. The development and regular use of prediction tools, which include relevant genetic and non-genetic risk factors, such as CanRisk, would likely facilitate the implementation and validation of the PRS as an additional risk marker in routine work-up. Although attractive, comprehensive risk prediction tools are complicated due to the non-availability and quality of data, and thus, models with only PRS (and FH) might be a feasible first step towards a more accurate CRC risk prediction for the general population.

## Prostate cancer

Prostate cancer (PC) is the most frequently diagnosed malignancy in men in Germany, with nearly 80,000 new cases reported in 2023. PC accounts for approximately 28 % of all male cancer diagnoses, and it represents the second leading cause of cancer-related death in men [58]. The lifetime risk of developing PC is approximately 14.2 %, and the relative 5-year survival rate is about 92 %, reflecting both the high incidence and the often indolent course of the disease [58]. Established PC risk factors include age, ancestry, FH, and genetic predisposition. Notably, PC is among the most heritable common cancers, with genetic susceptibility arising from both rare, moderate- to high-penetrance variants and a large number of common low-risk variants [49]. Comparative analyses indicate that the relative impact of PRS on PC risk is particularly pronounced compared to other common cancers such as BC or CRC [18].

Over the past decade, GWAS have identified hundreds SNPs associated with PC risk, underscoring the highly polygenic architecture of the disease [8, 61, 69]. Multiple studies have consistently demonstrated that PRS can stratify men across a broad spectrum of lifetime risk, with individuals in the highest percentiles exhibiting a two- to four fold increased risk compared to the population average [62]. As the PRS in monogenic BC and CRC, PRS in PC act as modifiers of penetrance in individuals with monogenic predisposition, such as carriers of pathogenic variants in *BRCA2* or other DNA repair genes [3, 36].

### Clinical utility and current perspective

At present, routine implementation of PRS in general population screening is not yet recommended in clinical guidelines, reflecting the need for further prospective validation and standardization [40]. There remains a central challenge in PC screening, namely the distinction between indolent and aggressive disease. However, in specialized settings – such as research-based screening programs – PRS already represent a valuable adjunct to established risk factors.

PRS-based risk stratification has been shown to improve the positive predictive value (PPV) of PSA testing, thereby enhancing the identification of clinically relevant disease and potentially reducing unnecessary diagnostic procedures [24]. PRS-guided selection of individuals for further diagnostic evaluation has already been demonstrated in prospective settings: In the BARCODE1 study, men in the highest PRS decile underwent PSA testing and MRI-based assessment, with biopsy performed according to study protocol. This approach resulted in a high detection rate of PC with a substantial proportion of clinically significant tumors [50]. Notably, a fraction of detected cancers occurred in individuals with low PSA levels and/or non-suspicious MRI findings, suggesting that such cases might have been missed under screening pathways based on PSA and MRI alone, and thus highlighting the potential complementary role of PRS [50]. Not designed as standalone diagnostic tools, PRS integration into multimodal risk assessment frameworks can help to enrich screening populations for individuals at higher risk of clinically significant cancer. Targeted screening approaches such as the PROFILE study further support the concept of combining genetic risk, FH and ancestry to identify high-risk populations for intensified screening strategies [7]. Based on large cohort datasets, the prostate-specific extension of the CanRisk framework, CanRisk-Prostate, enables estimation of short- and medium-term risk (e.g., 5- and 10-year risk) and offers a clinically interpretable output for patient counseling. Validation studies have demonstrated good calibration between predicted and observed PC incidence, supporting the robustness of this approach [53].

### Perspective

PRSs represent a significant advancement in the genetic stratification of PC risk and have reached a level of scientific maturity that supports their integration into multifactorial models such as CanRisk-Prostate. This is further supported by accumulating evidence demonstrating the performance of PRS across diverse ancestry groups, with PC and also BC representing some of the most advanced examples of clinical translation. Ongoing initiatives such as the German INTEGRATE study, funded by the German Cancer Aid, aim to further translate PRS into clinical practice by systematically evaluating their implementation within structured risk assessment frameworks and real-world patient cohorts.

## Other cancers including ovarian cancer

In the meantime, PRS were also developed and explored for other, relatively common tumour types such as ovarian [10], pancreatic [68], lung [75], thyroid [14], bladder [52], kidney [72], testis [43], and endometrial cancer [70]. The results of PRS-only or mixed model studies mirror largely the findings in the above mentioned tumors, in which a cancer-specific PRS was shown to be an independent risk factor, which may be associated with early-onset disease, and the combined analysis with modifiable clinical risk factors and FH result in additive effects with significant improvement in risk discrimination / predictive performance. Interestingly, some rare cancers such as intestinal carcinoids, testicular tumors, bone and thyroid cancer present with a relatively high familial risk in the absence of PGV in known high-penetrant genes, which may point to a stronger polygenic component than expected (Fig. 4) [20, 76].

Nonetheless, the clinical utility of an integrated PRS-based risk prediction has not been proven. Since most of these cancers are not subject of specific national surveillance programmes for the general population and no kind of specific risk stratification is established so far, they currently seem to be not the first choice for the implementation of a PRS-based risk asessment in routine clinical care.

Risk factors of high-grade serous adenocarcinoma, the most common subtype of ovarian cancer (OC), include age, obesity, and nulliparity among others. Around a quarter of OC are inherited, caused by PGV in genes underlying HBOC or Lynch syndrome (*BRCA1*/*2*, *RAD51C, RAD51D*, *BRIP1,* *PALB2,* MMR genes). An OC-based 36 SNP PRS associated with non-mucinous epithelial OC [10] has been implemented in the CanRisk tool (BOADICEA) [38]. Similar to the BC BOADICEA model, performance was best, when all risk factors were considered (AUC = 0.68, [73]). A lifetime cumulative OC risk of 3–4 % has been suggested as a threshold for risk-reducing salpingo-oophorectomy [41]. However, since preventive strategies (screening and prophylactic bilateral salpingo-oophorectomy) are not yet fully aligned with individualized OC risk estimates, the OC PRS’ clinical utility remains unclear at this stage.

## Challenges and limitations

Remarkably, the relative contribution of the polygenic background compared to other risk factors varies significantly among tumors and studies. While in some cancers, the genetic risk seems to exceed modifiable exposures (by far), such as colon, prostate, and thyroid cancers, leukemia and testis, other malignancies are characterized by a notably low portion, such as endometral and lung cancer (Fig. 4) [29, 76, 77]. It was suggested, that the likelihood to identify PGV in novel moderate and high-penetrant genes might be especially promising in tumor affected individuals with a low PRS.

A portion of the high heterogeneity observed even within PRS-only and mixed model studies, respectively, can be attributed to varying study designs, population characteristics, clinical risk factor selection, and the number of SNPs included in each model. Further research is needed to identify a comprehensive set of risk SNPs, standardize PRS construction, enhance accuracy, incorporate rare genetic variants and to provide more evidence of the clinical utility in different scenarios.

In addition, ethnic background significantly influences prediction accuray as outlined in articles Schumacher et al. “Polygenic risk scores in clinical applications – opportunities and challenges” as well as Klinkhammer et al. “An Introduction to Polygenic Scores – Methodological Basics and Recent Advances. The GWAS datasets underlying PRS models mainly comprises individuals of European ancestry with variation in PRS distribution even between European countries [74]. While studies have shown similar performance in Asian populations [21], transferability to other populations is generally limited, e.g. due to differences in allele frequencies [47]. In the multifactorial risk model BOADICEA/CanRisk, ancestry-specific PRS have now been implemented, enabling PRS-based risk prediction across populations. However, further efforts to develop ancestry-specific PRS and prospective validation studies are highly warranted given the potential of aggravating health inequities [16, 45].

## Conclusions and outlook

PRSs have emerged as a powerful and independent tool for refining cancer risk prediction across both hereditary and sporadic settings. As shown for breast, colorectal, prostate and several other malignancies, PRS enhance individual risk stratification beyond traditional risk factors. Integrating PRS with clinical and genetic risk determinants enables more precise identification of high- and low-risk individuals, thereby supporting tailored diagnostic and preventive strategies. This seems to be feasible in particular for cancers where clinically actionable risk thresholds already have been defined. While multifactorial tools such as CanRisk have demonstrated the practical value of incorporating PRS alongside clinical variables, prospective studies are still required to confirm their clinical utility, cost-effectiveness, and patient acceptance across populations. Importantly, evidence for a measurable clinical benefit on hard endpoints, such as a reduction on cancer incidence through primary prevention and mortality, remains limited to date. In BC feasibility of risk-adapted screening has been demonstrated in studies such as the WISDOM study; however, evidence of improved clinical outcomes is still lacking, and potential trade-offs, including increased biopsy rates, have been reported.

Despite their promise, significant challenges remain before PRS can be broadly applied in medical practice. Standardization of PRS construction, inclusion of ancestry-specific data, harmonization of reference datasets, and development of clear, evidence-based clinical recommendations are essential next steps. As ongoing implementation studies continue to generate real-world data, PRS are expected to become an important component of individualized risk management.
